# Sleep Deprivation During Memory Consolidation, but Not Before Memory Retrieval, Widens Threat Generalization to New Stimuli

**DOI:** 10.3389/fnins.2022.902925

**Published:** 2022-05-19

**Authors:** Eugenio Manassero, Alessandra Giordano, Erika Raimondo, Alessandro Cicolin, Benedetto Sacchetti

**Affiliations:** ^1^Department of Neurosciences “Rita Levi Montalcini”, University of Turin, Turin, Italy; ^2^Sleep Disorder Center, Department of Neurosciences “Rita Levi Montalcini”, University of Turin, Turin, Italy

**Keywords:** threat generalization, sleep deprivation, consolidation, retrieval, threat memory

## Abstract

Past aversive experiences shape our ability to deal with future dangers, through the encoding of implicit and explicit memory traces and through the ability to generalize defensive reactions to new stimuli resembling learned threats. Numerous evidence demonstrate that sleep is important for the consolidation of memories related to threatening events. However, there is a lack of studies examining the effects of sleep deprivation on the retrieval of consolidated threat memories, and previous studies on the role of sleep in threat generalization have produced mixed results. To address these issues, here we adopted a differential threat conditioning and a delayed (second half of the night) sleep deprivation during the first or the seventh night after learning. We found no effects of sleep deprivation on either implicit or explicit threat memories, regardless of its occurrence timing. Conversely, implicit but not explicit responses to novel cues similar to a learned threat displayed a widened generalization pattern, but only if sleep deprivation took place during the first night after conditioning and not if it occurred during the seventh night after conditioning. Therefore, we propose that sleeping after exposure to danger may support optimal implicit discrimination processes to evaluate new signals in the future and that even a brief period of sleeplessness may widen threat generalization to new stimuli, which is a hallmark of several threat-related disorders.

## Introduction

During our life, we constantly encode new information. By collecting and storing data about relevant episodes, our brain is able to formulate predictions about new incoming eventualities. In this scenario, dangerous encounters in the past assume an adaptive role in shaping our ability to deal with potential threats in the future. This knowledge about threatening events is embedded into emotional memories that, following a process of consolidation (McGaugh, 2000), integrate and expand our behavioral repertoire allowing us to react quickly and efficiently when encountering these situations again (DiFazio et al., 2022). To accomplish the consolidation of these memories, complex and selective offline processes occur during post-learning sleep (Diekelmann and Born, 2010; Goldstein and Walker, 2014), when memory representations are reactivated and reorganized (Hennevin et al., 2007). Indeed, previous studies indicate that sleep, and especially rapid eye movement (REM) sleep, may be critical for the consolidation of memories about fearful stimuli (Menz et al., 2013, 2016; Marshall et al., 2014; Spoormaker et al., 2014; Genzel et al., 2015; Pace-Schott et al., 2015). This evidence supports the proposed notion that REM sleep may provide overnight restoring functions that allow the brain to detect learned threatening cues (Goldstein and Walker, 2014). Threatening events are processed by different neural systems within the human brain (Squire, 2004; LaBar and Cabeza, 2006). The implicit system encodes the unconscious-emotional valence and is mediated by complex neural networks encompassing the amygdala (Phelps and LeDoux, 2005) and sensory cortices (Concina et al., 2019), whereas the explicit system encodes the conscious-episodic aspects and is mediated by hippocampal and medial temporal lobe (MTL) networks (Mayes et al., 2007). According to a large body of research, sleep seems to be differentially involved in the modulation of these memory systems (Maquet, 2001; Fischer et al., 2006; Diekelmann and Born, 2010; Rieth et al., 2010; Weber et al., 2014).

However, adaptation to a complex ecological niche does not only demand organisms to learn what is dangerous from experience, but also to calibrate mechanisms to generalize the prompted reactions to new stimuli resembling previously learned threats and to discriminate what is dangerous from what is not (Dunsmoor and Paz, 2015; Concina et al., 2018, 2021; Grosso et al., 2018). We previously observed a sharp dissociation between implicit-unconscious and explicit-conscious recognition patterns toward new incoming stimuli, supporting the idea that multiple systems mediate threat generalization and the prediction of possible dangers (Manassero et al., 2019). Failures of these strategies or dysregulations of the underlying neural mechanisms may result in maladaptive behaviors and anxiety or post-traumatic stress disorders (PTSDs) (Dunsmoor and Paz, 2015). Notably, comorbidities between these psychopathological instances and sleep disorders can be observed in clinical practice (Cox and Olatunji, 2016; Colvonen et al., 2019). However, to date only a few studies (Kuriyama et al., 2010; Davidson et al., 2015, 2018; Goldstein-Piekarski et al., 2015) have examined how sleep and sleep deprivation may modulate the generalization of defensive responses toward new and not previously experienced stimuli, generating mixed and inconsistent results (Tempesta et al., 2018).

Furthermore, after being consolidated, a memory trace can be reactivated during the recall phase, which mostly takes place in the awake state of consciousness. Although some studies reported how sleep deprivation may impair emotional memory retrieval in animals (Patti et al., 2010; Fernandes-Santos et al., 2012; Montes-Rodríguez et al., 2019), to our knowledge no study has been so far conducted to investigate the effects of sleep deprivation on the retrieval of consolidated threat memories as well as on threat generalization in humans.

To disentangle these unsolved queries about sleep functions in the fear domain, in this study we tested two related questions: (i) whether and how sleep deprivation may interfere with either immediate memory consolidation or remote memory retrieval and affect either implicit or explicit threat memories and (ii) whether and how sleep deprivation may interfere with either immediate memory consolidation or remote memory retrieval and affect the generalization of defensive responses either at the implicit or explicit level.

## Materials and Methods

### Participants

All participants (*n* = 108) were healthy individuals (mean age: 21.95 ± 3.51 SD, 70 females, and 38 males) with no history of psychiatric disorders, sleep disorders, neurological illnesses, cardiovascular diseases, and illegal drug use. During the pre-experimental screening phase, each recruited volunteer was administered with: (1) the State-Trait Anxiety Inventory Form Y (STAI-Y, Spielberger et al., 1983; Pedrabissi and Santinello, 1989), and who showed a score >80 in the sum of the two subscales (State + Trait anxiety) were not included in the sample; (2) the Beck Depression Inventory (BDI, Beck et al., 1961), and who scored >13 were excluded from the sample; (3) Pittsburg Sleep Quality Index (PSQI, Buysse et al., 1989), with an inclusion cut-off equal to 5; (4) Epworth Sleepiness Scale (ESS, Johns, 1991): cut-off = 10; (5) Insomnia Severity Index (ISI, Bastien et al., 2001), cut-off = 10; (6) Stop-Bang Questionnaire (Boynton et al., 2013), cut-off = 2; (7) Morningness-Eveningness Scale short version (Natale et al., 2006), and who obtained a “morning” or “evening” chronotype were not included in the sample; (8) Restless Legs Syndrome (RLS) criteria^1^, and who answered “yes” to one or more questions were not included in the sample. Musicians and individuals who reported a past or current musical training were not included in the sample (see Table 1 for all groups’ descriptive, experimental, and clinical data). After this preliminary phase, participants were randomly assigned to one of the six experimental conditions. We discarded two participants because of completely flat skin conductance responses (SCRs), and thirteen participants because they failed to accomplish the sleep deprivation protocol, leaving a total of 93 participants. Each participant provided written informed consent after receiving a complete description of the experimental procedures. All experimental procedures were performed in accordance with the ethical standards of the Declaration of Helsinki and were approved by the Bioethics Committee of the University of Turin.

**TABLE 1:** Experimental groups’ descriptive, experimental, and clinical data.

Group	*N*	Sex	Age	US (mA)	US rating	STAI-Y State (S1)	STAI-Y State (S2)	STAI-Y Trait	BDI	MEQ	ISI	ESS	PSQI	SBQ	RLS
IMPLICIT TASK															
CTRL	16	10F 6M	20.73 ± 2.31	3.92 ± 2.57	5.81 ± 1.61	32.38 ± 5.12	28.50 ± 7.13	35.94 ± 6.30	5.63 ± 4.24	14.75 ± 1.81	4.56 ± 2.45	5.75 ± 2.57	3.81 ± 1.38	0.81 ± 0.75	0.00 ± 0.00
DSDc	16	10F 6M	21.66 ± 4.41	4.16 ± 1.55	6.03 ± 1.42	31.50 ± 6.06	32.38 ± 5.80	36.69 ± 5.49	6.81 ± 4.81	14.25 ± 2.79	4.19 ± 3.12	6.25 ± 2.49	3.38 ± 1.20	0.69 ± 0.60	0.00 ± 0.00
DSDr	16	10F 6M	24.04 ± 4.13	5.47 ± 2.48	5.63 ± 1.36	31.38 ± 4.40	30.94 ± 6.03	34.81 ± 5.60	5.13 ± 4.33	13.50 ± 2.83	5.06 ± 3.99	5.81 ± 3.08	4.13 ± 1.50	1.00 ± 0.89	0.00 ± 0.00
EXPLICIT TASK															
CTRL	15	9F 6M	21.93 ± 3.17	5.47 ± 2.64	5.70 ± 1.50	30.80 ± 3.36	31.67 ± 6.59	34.60 ± 3.89	5.20 ± 3.57	15.07 ± 2.71	2.87 ± 2.03	5.00 ± 3.36	3.80 ± 1.47	0.60 ± 0.63	0.00 ± 0.00
DSDc	15	9F 6M	21.28 ± 3.53	5.09 ± 2.78	5.57 ± 1.40	32.87 ± 5.49	30.73 ± 5.73	38.67 ± 4.89	5.93 ± 3.03	14.93 ± 3.81	3.87 ± 3.34	6.13 ± 3.50	3.47 ± 1.51	1.00 ± 0.76	0.00 ± 0.00
DSDr	15	9F 6M	23.39 ± 3.40	4.44 ± 2.84	5.60 ± 1.23	30.07 ± 5.87	30.80 ± 5.92	37.13 ± 7.11	5.40 ± 2.80	15.00 ± 3.36	3.60 ± 2.44	5.07 ± 3.28	4.00 ± 2.10	1.00 ± 0.76	0.00 ± 0.00

*The table reports, for each experimental condition: sample size (N), sex distribution (F, female; M, male), mean age, US current intensity (mA), post-conditioning US rating, STAI-Y, State-Trait Anxiety Inventory Form Y; State subscale score during session 1 (S1) and session 2 (S2), and Trait subscale score, BDI, Beck Depression Inventory score; MEQ, Morningness-Eveningness Scale short version score; ISI, Insomnia Severity Index score; ESS, Epworth Sleepiness Scale score; PSQI, Pittsburg Sleep Quality Index score; SBQ, Stop-Bang Questionnaire score; and RLS, Restless Legs Syndrome diagnostic criteria score. All data are mean ± standard deviation.*

### Experimental Outline

To explore the effects of sleep deprivation on either implicit or explicit defensive responses, in the first experimental session (day 1) we implemented a differential auditory threat conditioning paradigm where participants learned to associate a tone (conditioned stimulus, CS+, 370 Hz, or 784 Hz, counterbalanced) with a mild electrical shock (unconditioned stimulus, US, individually calibrated intensity) and another tone (non-reinforced stimulus, CS−, 784 Hz or 370 Hz, counterbalanced) with no shock. Since we intended to evaluate the memories related to threatening and safe stimuli as well as the threat generalization toward new stimuli, 1 week after threat learning (day 8) we tested participants’ implicit and explicit recognition patterns toward the learned cues as well as toward new tones. To this purpose, they were randomly assigned to six different experimental conditions. Three groups underwent an *implicit* two-alternative forced-choice (2AFC) test, in which subjects were presented with a pseudorandom sequence of tone pairs, each composed of a conditioned stimulus (CS+ or CS−) and a new stimulus, harmonically similar and higher-pitched than the CS+ (new stimulus similar to the CS+, NS+, 466 Hz or 1,046 Hz, counterbalanced depending on the frequency of the CS+) or than the CS− (new stimulus similar to the CS−, NS−, 1,046 Hz or 466 Hz, counterbalanced depending on the frequency of the CS−) (Manassero et al., 2019). In order to assess implicit defensive reactions, electro-dermal skin conductance responses (SCRs) were recorded to characterize the event-related autonomic reactivity. Three other groups performed an *explicit* 2AFC task, in which participants heard the identical sequence of CS-NS pairs used during the implicit test, and they had to identify which stimulus of each pair was the one previously paired with the US (CS+) or the one previously learned as not associated with the US (CS−). Subjects were also asked to provide subjective confidence feedback for each choice, utilizing an analog scale ranging from 0 (completely unsure) to 10 (completely sure). No US shocks were delivered during the implicit and explicit tests (Manassero et al., 2019).

**FIGURE 1:**
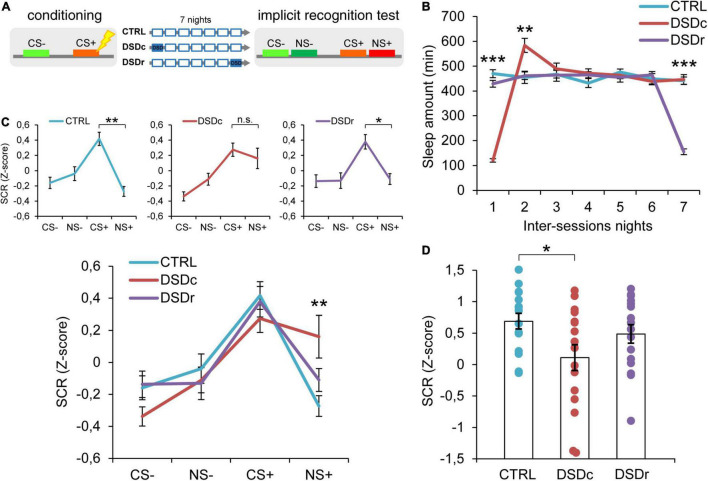
Effects of DSD on implicit threat memories, safety memories, and threat generalization to new stimuli. **(A)** Schematic diagram depicting the experimental design. Participants underwent a differential threat conditioning in which a conditioned tone (CS+) was paired with a mild electrical shock (US) and a non-reinforced tone (CS–) was never paired with the US. One group of participants (CTRL, *n* = 16) was allowed to regularly sleep during the seven nights between the sessions. The other two groups were sleep-deprived during the night after the first session (DSDc, *n* = 16) or during the night before the second session (DSDr, *n* = 16). One week after the conditioning session, subjects underwent an implicit 2AFC recognition task during which they were presented with tone pairs composed of a conditioned stimulus (CS– or CS+) and a new stimulus similar to the CS– (NS–) or the CS+ (NS+), while SCRs were recorded. **(B)** Actigraphically controlled sleep distributions of each group over the seven nights separating the experimental sessions. **(C)** Implicit reactions to the learned threatening (CS+) and safety-signaling (CS–) stimuli were comparable among conditions, whereas implicit reactions to the new stimulus (NS+) were higher in the DSDc group than in the CTRL group. Implicit reactions to the NS+ were weaker than those to the CS+ in the CTRL and the DSDr but were similar to those to the CS+ in the DSDc group. **(D)** Specificity of defensive responses (CS+ minus NS+) was higher in the CTRL group than in the DSDc group. *P < 0.05, **P < 0.01, ***P < 0.001. All data are mean and SEM. 3 × 7 mixed ANOVA followed by Bonferroni corrected simple main effect analyses **(B)**; 3 × 4 mixed ANOVA followed by Bonferroni corrected simple main effect analyses **(C)**; One-way ANOVA followed by Bonferroni-adjusted *post hoc* comparisons **(D)**.

**FIGURE 2:**
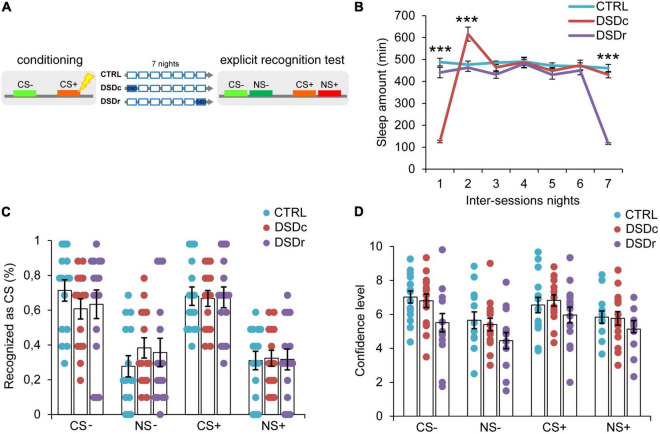
Effects of DSD on explicit threat memories, safety memories, and threat generalization to new stimuli. **(A)** Schematic diagram depicting the experimental design. Participants underwent a differential threat conditioning in which a conditioned tone (CS+) was paired with a mild electrical shock (US) and a non-reinforced tone (CS–) was never paired with the US. One group of participants (CTRL, *n* = 15) was allowed to regularly sleep during the seven nights between the sessions. The other two groups were sleep-deprived during the night after the first session (DSDc, *n* = 15) or during the night before the second session (DSDr, *n* = 15). One week after the conditioning session, subjects underwent an explicit 2AFC recognition task during which they were presented with tone pairs composed of a conditioned stimulus (CS– or CS+) and a new stimulus similar to the CS– (NS–) or the CS+ (NS+) and they were asked to recognize the CS– and the CS+, providing a confidence level for each choice. **(B)** Actigraphically controlled sleep distributions of each group over the seven nights separating the experimental sessions. **(C)** Explicit recognition patterns for correct (CS– and CS+) and incorrect (NS– and NS+) choices were comparable between groups. **(D)** Confidence ratings for correct (CS– and CS+) and incorrect (NS– and NS+) choices were similar amongst conditions. ***P < 0.001. All data are mean and SEM. 3 × 7 mixed ANOVA followed by Bonferroni corrected simple main effect analyses **(B)**; 3 × 4 mixed ANOVA **(C,D)**.

To determine the potential impact of sleep deprivation, four groups of participants underwent a one-night delayed sleep deprivation protocol (DSD, 75%, individually calibrated based on daily self-reported sleep diaries), while they were actigraphically monitored. Since REM sleep prevails during the second half of the night (Diekelmann and Born, 2010), DSD took place after an initial 25% sleeping period. To selectively interfere with immediate memory consolidation or remote memory retrieval, two experimental groups performed the DSD during the first night following the learning session (i.e., early memory consolidation, DSDc conditions), and they were respectively, tested with the implicit (*n* = 16) and the explicit (*n* = 15) recognition tasks. Two other experimental groups performed the DSD during the seventh night from learning (i.e., the night before retrieval, DSDr conditions), and they performed the implicit (*n* = 16) and the explicit (*n* = 15) tests. As control conditions (Ctrl), the last two groups regularly slept during all the seven nights between the sessions and were also tested on the implicit (*n* = 16) and the explicit (*n* = 15) tasks (Figures 1A, 2A).

### Auditory Stimuli

Auditory stimuli were pure sine wave tones with oscillation frequencies of 370, 466, 784, and 1,046 Hz, lasting 6 s with onset/offset ramps of 5 ms. Tones were digitally generated using Audacity 2.1.2 software (Audacity^®^ freeware) and binaurally delivered through headphone speakers (Beyerdynamic DT770 Pro) at ∼50 dB intensity. Experiments were conducted in a dimly lit room, and all experimental scenarios were controlled by Presentation^®^ 21.1 software (NeuroBehavioral Systems Inc., Berkeley, CA, United States).

### Unconditioned Stimulus Calibration Procedure

Before starting with the calibration procedure, systolic and diastolic blood pressure was measured to prevent possible hypoarousal reactions caused by basal hypotension. The unconditioned stimulus (US) consisted of a mild electrical shock (train pulse at 50 Hz lasting 200 ms, with a single pulse duration of 1,000 μs) generated with a direct current stimulator (DS7A Constant Current Stimulator, Digitimer). Impulses were delivered through a bar stimulating electrode connected by a Velcro strap on the upper surface of the dominant hand’s index finger. The electrical stimulation intensity was individually calibrated through a staircase procedure (Cornsweet, 1962), starting with a low current near the perceptible tactile threshold (∼0.5 mA). Participants were asked to rate the aversiveness of each train-pulse on a scale ranging from 0 (not painful at all), 1 (pain threshold) to 10 (highly painful if protracted in time). At the end of the procedure, the US amplitude was set at the current level (mA) corresponding to the mean rating of “7” on the subjective analog scale.

### Pre-conditioning

This phase consisted of the presentation of 4 stimuli: 2 CS− (784 Hz or 370 Hz, counterbalanced) and 2 CS+ (370 Hz or 784 Hz, counterbalanced) tones with an inter-trial-interval (ITI) of 24 s, in absence of any US. At the end of this phase, participants were asked to confirm whether the tones were easily audible but not too loud or annoying.

### Conditioning

After a 5-min resting period, participants underwent a differential threat conditioning, which consisted of the presentation of 30 stimuli: 15 CS+ (370 Hz or 784 Hz, counterbalanced) and 15 CS− (784 Hz or 370 Hz, counterbalanced) in a pseudorandom sequence, with an inter-trial-interval (ITI) of 24 s. The CS+ co-terminated with the US 12 times (80% reinforcement rate), while the CS− was never paired with the US. Subjects were not informed about any possible CS-US contingency. To validate the unpleasant nature of the emotional learning, immediately following this phase subjects rated the aversiveness of the USs experienced during the conditioning session. The rating was performed using the same analog scale as in the pre-conditioning calibration procedure (see Table 1 for all groups’ US current intensity and post-conditioning US analog ratings).

### Sleep Deprivation

Participants were preliminarily asked to complete a self-assessment of their sleep using a sleep diary to determine their habitual sleep-wake cycle over 1 week. Based on the data collected from each diary, during the night after the first experimental session (implicit and explicit DSDc groups) or the night before the second experimental session (implicit and explicit DSDr groups), subjects underwent a delayed sleep deprivation (DSD) of 75% of their average total sleep time (TST), as follows: the average total sleep time calculated over the 7 days preceding the experimental session was determined and 75% of the total sleep time was subtracted (e.g., if one subject’s average TST during the week preceding the experimental session was 7 h, the scheduled TST was 1 h and 45 min). A bedtime and a wake-up time (after an initial 25% of sleep) were then set based on the individual subjects’ average bedtime habits, resulting from the sleep diary. TST has been reduced by 75% since, according to several studies (Groeger et al., 2004; Luckhaupt et al., 2010; Lo et al., 2012), 25% of sleep is approximately the minimum sleep duration reported by a large segment of the working population. Subjects were not allowed to assume stimulants (such as caffeine) during the sleep deprivation night. Actual sleep during the experimental scheduled night was monitored by actigraphic recording (Actiwatch Spectrum Plus, Philips Respironics, Murrysville, PA, United States). An actigraph is a small, light-weight wrist-worn computerized device with the ability to measure, through an accelerometer, the rest-activity patterns continuously over long periods. Mathematical algorithms are then applied to these data to estimate wakefulness and sleep (Smith et al., 2018). Data were analyzed through Actiware 6.0.9 (Philips Respironics, Murrysville, PA, United States).

### Two-Alternative Forced-Choice Recognition Test

This procedure involves the presentation of two stimuli on each trial and the subject chooses the one that was previously encoded (i.e., the first or the second one). A 2AFC design was preferred over a new-old paradigm, which involves one single stimulus on each trial and the subject judges whether the stimulus has been previously encoded (old), or whether it is new. Our choice was motivated by the evidence that a 2AFC task improves recognition performance and discourages response biases such as the familiarity-based decision bias, namely the heuristic to endorse novel cues as “old” when their familiarity is high (Macmillan and Creelman, 2004).

Throughout the testing phase, the stimulating electrode was kept attached as in the conditioning phase to create the expectation to receive the US (Ameli et al., 2001). Differently from other generalization paradigms which involve the delivery of the US to prevent extinction (Lissek et al., 2013; Holt et al., 2014; Onat and Büchel, 2015; Dunsmoor et al., 2017), here no shocks were delivered, i.e., the CS+ was never paired with the US, to avoid any reacquisition effect (Manassero et al., 2019).

After a 5-min resting period, participants underwent a two-alternative forced-choice (2AFC) task, which consisted of the presentation of 20 pairs of auditory stimuli, each composed of a conditioned stimulus (CS− or CS+) and a new stimulus similar to the CS− (NS−, 1,046 Hz or 466 Hz, counterbalanced) or to the CS+ (NS+, 466 Hz or 1,046 Hz, counterbalanced) in a pseudorandom sequence: 5 × CS− vs. NS−, 5 × NS− vs. CS−, 5 × CS+ vs. NS+, 5 × NS+ vs. CS+. On each trial, the two stimuli were presented with an intra-trial-interval of 1000 ms. After 5 s from the pair offset, a 60-s auditory interference (see next section) and a 24-s silent ITI occurred. In the implicit test, SCRs were recorded throughout this phase. In the explicit test, participants were explained that in each couple of sounds there was a tone that they had heard in the first session and a new tone. Participants were then instructed to recognize and verbally identify which one (the first or the second) was the tone heard in the first session, paired (CS+) or not paired (CS−) with the US-shock. Participants were further asked to verbally provide a confidence rating about each response, on a scale from 0 (completely unsure) to 10 (completely sure). No feedback was supplied.

### Auditory Working Memory Interference

When hearing a serial sequence of tones subjects can actively take advantage of a pitch comparison mechanism due to the auditory working memory (AWM) rehearsal process (Keller et al., 1995; Kumar et al., 2016). In our testing protocol, if participants were not prevented from rehearsing during the inter-trial-interval, each response (except for the first one) might be affected by the sensory comparison of each pair of tones with the previous one in the sequence, thus introducing cognitive biases in the recognition process. Given that a method to interfere with the rehearsal process is filling the inter-trial-interval with a series of additional tones (Deutsch, 1970; Pechmann and Mohr, 1992), we created an auditory retroactive interference to prevent possible cognitive biases during the recognition test. The interference consisted of 60 s of 10-s mixed samples of pop music (Manassero et al., 2019).

### Psychophysiological Recording and Analysis

Event-related skin conductance responses (SCRs) were used as an implicit index of defensive responses. To record the autonomic signal, two Ag-AgCl non-polarizable electrodes filled with isotonic paste were attached to the index and middle fingers of the non-dominant hand by Velcro straps. The transducers were connected to the GSR100C module of the BIOPAC MP-150 system (BIOPAC Systems, Goleta, CA, United States) and signals were recorded at a channel sampling rate of 1,000 Hz. SCR waveforms were analyzed offline using AcqKnowledge 4.1 software (BIOPAC Systems, Goleta, CA, United States). Each SCR was evaluated as event-related if the trough-to-peak deflection occurred 1–6 s after the stimulus onset, the duration was comprised between 0.5 and 5.0 s, and the amplitude was greater than 0.02 micro siemens (μS). Responses that did not fit these criteria were scored as zero. Because the implicit test was configured as 2AFC with a 1-s ITI, the range of the analysis was restricted to 1–7 s following the onset of the first stimulus of the pair. That is, given the sequence of 6 s (1st tone), 1 s (intra-trial-interval), and 6 s (2nd tone) which defined the structure of each pair, a temporal cut-off was established upon the onset of the second stimulus of the pair, to avoid summing effects in the event-related responses. Raw SCR data of each subject were standardized through a *Z*-score transformation (Ben-Shakhar, 1985; Braithwaite and Watson, 2015) and averaged by stimulus type (CS−, NS−, CS+, and NS+).

### Statistical Analyses

Since all variables passed the D’Agostino-Pearson omnibus normality test, parametric statistics were adopted in each experiment. To test the between-groups differences in post-conditioning US ratings for implicit and explicit conditions, we performed two one-way ANOVA models. To compare the inter-sessions sleep amount of each group in the implicit and explicit conditions, we computed two 3 × 7 mixed-design ANOVAs with Group (CTRL, DSDc, and DSDr) as between-subjects variable and Time (Night 1–7) as within-subjects variable. Bonferroni adjustment was applied for simple main effects analyses. To test the main effects of group and tone, and the interaction effect in the implicit (autonomic data) and explicit (choice rates and confidence levels) tests, we employed three 3 × 4 mixed-design ANOVAs with Group (CTRL, DSDc, and DSDr) as between-subjects variable and Tone (CS−, NS−, CS+, and NS+) as within-subjects variable. Bonferroni adjustment was applied for simple main effects analyses. For each mixed ANOVA model, we assessed the Sphericity assumption through Mauchly’s Test. Where it was violated, we applied the Greenhouse–Geisser correction accordingly. To assess between-groups differences in the specificity index, we calculated a one-way ANOVA model with Bonferroni-adjusted *post hoc* comparisons. The null hypothesis was rejected at *P* < 0.05 significance level. All statistical analyses were performed using SPSS Statistics 22 (IBM) and Prism 6.05 (GraphPad).

## Results

### Homogeneity of Experimental Groups in Unconditioned Stimulus Ratings and Sleep Distribution

To ensure a between-groups comparability in the emotional learning experience, we performed two one-way ANOVA models on post-conditioning US ratings (see Table 1), and we found no significant differences either for implicit [*F*_(2,45)_ = 0.3071, *P* > 0.05, η_p_^2^ = 0.0135] or explicit [*F*_(2,42)_ = 0.03795, *P* > 0.05, η_p_^2^ = 0.0018] conditions. To compare the sleep amount of each group between the experimental sessions, we computed two 3 × 7 mixed ANOVA models on the quantifications of sleep resulting from the actigraphically controlled sleep diaries. For the implicit conditions, the analysis showed a significant main effect of group [*F*_(2,45)_ = 4.701, *P* = 0.014, η_p_^2^ = 0.173], a significant main effect of time [*F*_(4.664,209.862)_ = 47.869, *P* < 0.001, η_p_^2^ = 0.515] and a significant group × time interaction [*F*_(9.327,209.862)_ = 44.897, *P* < 0.001, η_p_^2^ = 0.666]. As expected, in the first night after learning, the DSDc group slept less than the other two conditions (*P* < 0.001 in both cases, Bonferroni corrected), while in the night before the test the sleep amount of the DSDr group was lower than that of the other two conditions (*P* < 0.001 in both cases, Bonferroni corrected). During the second night after learning, the DSDc group slept more than the Ctrl (*P* = 0.001) and the DSDr (*P* = 0.002) groups, likely due to a sleep recovery effect. No between-groups differences were found in the other nights (*P* > 0.05) (Figure 1B). For the explicit conditions, we found similar results [main effect of group: *F*_(2,42)_ = 15.831, *P* < 0.001, η_p_^2^ = 0.430; main effect of time: *F*_(4.655,195.529)_ = 49.076, *P* < 0.001, η_p_^2^ = 0.539; group × time interaction: *F*_(9.311,195.529)_ = 42.875, *P* < 0.001, η_p_^2^ = 0.671]. The DSDc group slept less than the other two conditions during the Night 1 (*P* < 0.001 in both cases, Bonferroni corrected) whereas the DSDr group slept less than the other two conditions during the Night 7 (*P* < 0.001 in both cases, Bonferroni corrected). Again, during the Night 2 the sleep amount of the DSDc group was higher than those of the other two conditions (*P* < 0.001 in both cases, Bonferroni corrected) and no differences were found in the other nights (*P* > 0.05) (Figure 2B).

### Effects of Delayed Sleep Deprivation on Implicit and Explicit Recognition of Learned Threatening and Safe Stimuli

To determine whether DSD affected implicit memories related to the threatening and safe cues, we performed a 3 × 4 mixed ANOVA model which revealed a not significant main effect of group [*F*_(2,45)_ = 1.09, *P* > 0.05, η_p_^2^ = 0.046], a significant main effect of tone [*F*_(3,135)_ = 18.30, *P* < 0.001, η_p_^2^ = 0.289] and a significant group × tone interaction [*F*_(6,135)_ = 2.217, *P* = 0.045, η_p_^2^ = 0.09]. Bonferroni corrected simple main effect analyses showed that participants successfully formed a fear memory, as SCR levels evoked by the CS+ were stronger than those evoked by the CS− in all groups (Ctrl: *P* < 0.001; DSDc; *P* < 0.001; DSDr: *P* = 0.001). Between-groups comparisons showed no effects of DSD on fear strength since all groups similarly reacted to the CS+ (*P* > 0.05 in all cases). Safety encoding was also not influenced by DSD, since reactions to the CS− were comparable among groups (*P* > 0.05 in all cases). Hence, our data suggest that DSD occurring during the first night or the seventh night after conditioning did not affect the autonomic reaction patterns toward learned threatening and safety-signaling stimuli (Figure 1C and Supplementary Figure 1).

Concerning the effects of DSD on explicit recognition patterns of the learned stimuli, a 3 × 4 mixed ANOVA model revealed a not significant main effect of group [*F*_(2,42)_ = 0.00, *P* > 0.05, η_p_^2^ = 0.00], a significant main effect of tone [*F*_(3,126)_ = 22.711, *P* < 0.001, η_p_^2^ = 0.351] and a not significant group × tone interaction [*F*_(6,126)_ = 0.413, *P* > 0.05, η_p_^2^ = 0.019]. Thus, we found no significant differences in the conscious identification of the CS+ (*P* > 0.05) and the CS− (*P* > 0.05) among conditions (Figure 2C). For confidence ratings provided by participants in the explicit task, a 3 × 4 mixed ANOVA model revealed a not significant main effect of group [*F*_(2,32)_ = 2.126, *P* > 0.05, η_p_^2^ = 0.117], a significant main effect of tone [*F*_(3,96)_ = 11.717, *P* < 0.001, η_p_^2^ = 0.268] and a not significant group × tone interaction [*F*_(6,96)_ = 0.674, *P* > 0.05, η_p_^2^ = 0.04]. The three groups were similarly confident when identified the CS+ (*P* > 0.05) and the CS− (*P* > 0.05) stimuli (Figure 2D). Therefore, our data indicate that DSD produced no effect on the participants’ ability to explicitly recognize learned threatening and safety-signaling stimuli as well as on their subjective confidence for explicit choices, regardless of the timing of its occurrence.

### Effects of Delayed Sleep Deprivation on Implicit and Explicit Reactions Toward New Stimuli

We then moved to explore how sleep-deprived and control participants implicitly reacted when exposed to previously unexperienced stimuli resembling threatening or safe ones. The Ctrl group exhibited a highly precise threat identification pattern, as the CS+ evoked higher SCRs than the NS+ (*P* = 0.001). Strikingly, the group deprived during the first night after conditioning (DSDc) showed a widened threat generalization, as the NS+ elicited similar SCRs to those triggered by the CS+ (*P* > 0.05). On the contrary, the group deprived during the seventh night after conditioning (DSDr) displayed an elevated specificity, as SCRs evoked by the CS+ were stronger than those evoked by the NS+ (*P* = 0.024). There were no differences in the evoked autonomic responses to the CS− and to the NS− in all groups (*P* > 0.05). Moreover, while autonomic reactions to the NS− were comparable among groups (*P* > 0.05 in all cases), SCRs evoked by the NS+ in the DSDc group were consistently stronger than those evoked by the NS+ in the Ctrl group (*P* = 0.007) and not different in other cases (*P* > 0.05) (Figure 1C and Supplementary Figure 1). To further characterize the level of threat generalization, we computed a specificity index by subtracting, for each subject, the mean response to the NS+ from the mean response to the CS+. A one-way ANOVA model determined significant between-groups differences [*F*_(2,45)_ = 3.291, *P* = 0.046, η_p_^2^ = 0.1276]. *Post hoc* Bonferroni-adjusted comparisons confirmed that specificity was significantly sharper in the Ctrl group than in the DSDc group (*P* = 0.045), while other comparisons were not significant (*P* > 0.05) (Figure 1D). Hence, we found that DSD provoked a widening of generalization of defensive reactions toward a never experienced stimulus (NS+) resembling the learned threatening cue (CS+), but only when it took place during the first night after learning and not when it occurred during the seventh night after learning (i.e., the night before retrieval). Conversely, we observed no effects of DSD when subjects implicitly responded to a novel stimulus (NS−) resembling a cue learned as safe (CS−), independently of its occurrence timing.

Explicit recognition patterns of the threatening stimulus (CS+) with respect to the new similar stimulus (NS+) resulted in a mean accuracy level of 68.0%, which was statistically different from the 32.0% of incorrect NS+ choices (*P* < 0.001, Bonferroni corrected). Participants were also able to precisely identify the safety-signaling cue (CS−) in 65.8% of cases, showing an accuracy rate that was significantly higher than 34.2% of incorrect NS− choices (*P* = 0.002, Bonferroni corrected). Misidentifications of both the NS+ and the NS− did not differ between conditions (*P* > 0.05 in all cases) (Figure 2C). Subjects reported higher confidence levels when they correctly identified the CS+ than when they misidentified the NS+ (*P* = 0.018), as well as they were more confident when they accurately recognized the CS− than when they incorrectly recognized the NS− (*P* = 0.001). Confidence levels did not differ between groups for both incorrect NS+ and NS− choices (*P* > 0.05) (Figure 2D). Therefore, our data indicated that DSD occurring during the first night or the seventh night from conditioning exerted no impact on participants’ explicit ability to detect learned threatening and safe stimuli discriminating them from new similar ones, thus revealing divergent DSD effects on explicit and implicit threat generalization.

## Discussion

In this study, we sought to test the effects of DSD on implicit and explicit memories related to threatening and safety-signaling stimuli, as well as on the generalization of implicit and explicit defensive reactions toward never experienced stimuli resembling learned threats or safety-signaling stimuli. Within a differential threat conditioning design, participants underwent a delayed sleep deprivation during the first night after learning or during the seventh night after learning (the night preceding the test), and then separately tested on their implicit and explicit recognition profiles. First, we observed that implicit and explicit memories related to threat and safe cues were not affected by DSD, neither if it took place during the first night nor the seventh night after learning. Second, implicit but not explicit reactions to new stimuli were shifted to a wider generalization when the DSD took place during the first night after learning but not when it was interposed during the seventh night after learning. Based on these findings, we propose that even a mild sleep deprivation that did not impair the memory traces may be sufficient to disrupt the specificity of reactions to new unexperienced stimuli, suggesting the idea that these mechanisms might be more sensitive to sleep alterations. This notion is relevant since threat overgeneralization has been recognized as a hallmark of several threat-related disorders, such as PTSD (Dunsmoor and Paz, 2015).

The over-time existence of memory implies distinct stages: encoding, consolidation, and retrieval (LaBar and Cabeza, 2006). Concerning the role of sleep and sleep deprivation in the consolidation of threatening memories, a previous study (Menz et al., 2013) reported increased SCRs to a learned safe stimulus but no differences to a learned threatening cue in the sleep-deprived group. In this study, the authors did not test defensive reactions to new stimuli and considered successful consolidation of threat memories as robust discrimination between learned threatening and safe stimuli. Based on this concept and the finding of stronger reactions to the CS− with unvaried reactions to the CS+, they showed that the consolidation of threat memories is related to the time spent in REM sleep. In a following study (Menz et al., 2016), the same authors used a split-night design and proposed a causal role of REM but not slow-wave sleep (SWS) in these processes. However, other works (Spoormaker et al., 2014) observed a REM-related reduction of defensive reactions to learned threats. In the current study, we did not detect any sleep-dependent alteration of implicit memories, in terms of higher or lower discrimination between learned danger and safety signals, possibly because our DSD protocol was too mild to disrupt the consolidation of the engrams. Importantly, all of these studies – including ours, used SCRs as the implicit index of differential responses evoked by the CS+ and CS−, while a recent investigation (Ojala and Bach, 2020) reported that pupil size and startle-eye blink responses appear to better discriminate CS+ and CS−, providing a higher validity in a threat learning paradigm. In the case of explicit memories, despite former studies revealing selective overnight enhancement of recognition accuracy (Drosopoulos et al., 2005) also for emotional material (Hu et al., 2006), here we did not find any DSD effects also on explicit recognition patterns. One possible explanation may rely on different susceptibilities to sleep interference of memory systems, where explicit processes may be more refractory to sleep restrictions. Indeed, some studies (Diekelmann et al., 2009) suggested that declarative memory may already take advantage of short (1–2 h, as in the case of the present study) sleep intervals, while non-declarative memory gains seem more dependent on the amount of sleep following the day after learning.

As far as we know, this is the first human study targeting the effects of DSD on the retrieval of consolidated threat memories. Here, we did not find any effect on either the autonomic reaction patterns or the cognitive identification of learned threatening and safe stimuli. Based on these findings, one may speculate that encountering a danger again, while being acutely sleep-deprived, may not imply alterations in the defensive reactions in humans. Alternatively, one may attribute this lack of effect to the adopted DSD paradigm. Indeed, one night of DSD might have been a too mild manipulation to produce a significant impairment of retrieval processes in the case of consolidated memories. In line with this idea, animal research demonstrated that a pre-test sleep deprivation impaired memory recall (Patti et al., 2010; Fernandes-Santos et al., 2012; Montes-Rodríguez et al., 2019), suggesting that sleep may be essential not only for encoding and consolidation but also for the retrieval of threat contextual memories. Future studies adopting total or chronic sleep deprivation will be necessary to disambiguate this possibility.

At variance with the lack of effects on memory consolidation and retrieval, we found that sleep deprivation broadened threat generalization of implicit reactions to novel stimuli. These data provide the new and important information that implicit discrimination of new stimuli may be more vulnerable to sleep deprivation than explicit discrimination and implicit or explicit memory. Generalization of defensive responses to new stimuli is an intrinsic property of animals -including humans- functioning, that allows protecting behaviors against new situations perceptually similar to known ones that predict adversity (Ghirlanda and Enquist, 2003; Dymond et al., 2015). It has been previously suggested that this ability may be conceptualized as an active process decoupled from perceptual processes (Onat and Büchel, 2015), and we previously uncovered a sharp dissociation between the implicit and the explicit reactions to new potential dangers (Manassero et al., 2019). In examining the relationship between sleep and generalization, it has been reported contradictory results (Tempesta et al., 2018). Sleep deprivation biased the discrimination of threatening from not threatening human faces, by shifting subjective ratings toward a higher categorization as threatening. Stimulus-evoked heart rate elevations were also altered by sleep deprivation, resulting in disrupted autonomic discrimination of threatening from non-threatening social stimuli (Goldstein-Piekarski et al., 2015). However, other investigations (Kuriyama et al., 2010; Davidson et al., 2018) yielded opposite results by providing evidence that sleep deprivation after exposure to aversive stimuli reduced threat generalization. One further work (Davidson et al., 2015) detected no significant differences between the awake group and sleep-controls in autonomic and subjective threat generalization tunings. A recent investigation (Lerner et al., 2021) using stimuli made by combinations of cues predicting aversive outcomes or safety, found opposing effects of REM sleep on recall and generalization. REM sleep impaired the ability to discriminate between encoded memories containing a combination of threat and safety signals but enhanced the ability to discriminate between new stimuli containing either threat or safety signals. In this framework, our data imply a DSD-dependent widening of implicit but not explicit generalization patterns to unexperienced stimuli resembling learned dangers. One possible way to conciliate our results with prior mixed findings consists of noting that our design was structured over 1 week and thus allowed us to separately target early consolidation and subsequent retrieval of consolidated memories. In this manner, resulting differences may be confined to specific memory epochs, and not to post-learning sleep deprivation.

Apparently at odds with our previous results reporting a threat-selective generalization of explicit responses after 24 h from learning (Manassero et al., 2019), we did not observe a comparable pattern at 1 week after conditioning. Indeed, participants of the control group correctly identified both the CS+ and the CS−, discriminating them from the NSs. On the other hand, implicit profiles with a 1-week retention interval were comparable to those we had found at 24 h following learning. One possible interpretation for this discrepancy may rely on the idea that the flexible and reversible mechanism we had hypothesized to underlie explicit processing may be important during the initial phases of consolidation, and over-time interactions between the two systems enable explicit tunings to converge with those of implicit dynamics.

Findings in this field are relevant for clinical implications since troubled sleep is commonly reported across anxiety and post-traumatic disorders (Kobayashi et al., 2007; Babson and Feldner, 2010; Soehner and Harvey, 2012; Cox and Olatunji, 2016). Nevertheless, the direction of this relationship is controversial, with some evidence (Pace-Schott et al., 2015) suggesting that trauma may be points of origin for sleep disturbances, and others (Bryant et al., 2010; Wright et al., 2011; Gehrman et al., 2013; Lerner et al., 2017) arguing that pre-existing sleep disorders may represent risk factors for the development of PTSDs. One may wonder whether it would be wiser to sleep or not to sleep in the aftermath of exposure to a natural disaster. According to some authors (Walker and van der Helm, 2009; Goldstein and Walker, 2014), REM sleep affords our ability to decouple the emotional charge from the memory, to achieve the strengthening of salient learned information and, in parallel, the depotentiation of the arousing charge that signaled alarm at encoding. Thus, sleep deprivation may block this adaptive removal of emotion from memory. In the view of other investigators (Kuriyama et al., 2010) post-traumatic insomnia may prevent threat generalization and PTSD onset. Based on the data of the current study, sleeping after exposure to danger may be protective against the overgeneralization of implicit alarm reactions, which has been qualified as a pathogenic marker of anxiety disorders and PTSD (Jovanovic et al., 2012; Dunsmoor and Paz, 2015).

Taken as a whole, our findings may contribute to elucidating the functions of sleep in the emotional domain, by providing support to the notion that implicit discrimination of new incoming stimuli may be more susceptible to sleeplessness than explicit discrimination and implicit or explicit memory. Following aversive events, sleep may promote optimal autonomic tunings to evaluate novel and previously unexperienced signals in the future.

## Data Availability Statement

The raw data supporting the conclusions of this article will be made available by the authors, without undue reservation.

## Ethics Statement

The studies involving human participants were reviewed and approved by the Comitato di Bioetica dell’Ateneo (University of Turin). The participants provided their written informed consent to participate in this study.

## Author Contributions

EM devised and carried out the experiments, analyzed the behavioral data, and wrote the manuscript. AG and ER carried out the experiments and analyzed the actigraphical data. AC devised the experiments and interpreted the data. BS devised the experiments, interpreted the data, and wrote the manuscript. All authors discussed the results and commented the manuscript.

## Conflict of Interest

The authors declare that the research was conducted in the absence of any commercial or financial relationships that could be construed as a potential conflict of interest.

## Publisher’s Note

All claims expressed in this article are solely those of the authors and do not necessarily represent those of their affiliated organizations, or those of the publisher, the editors and the reviewers. Any product that may be evaluated in this article, or claim that may be made by its manufacturer, is not guaranteed or endorsed by the publisher.

## Footnotes

1
https://irlssg.org/diagnostic-criteria/

## References

[B1] Ameli R. Ip C. Grillon C. (2001). Contextual fear-potentiated startle conditioning in humans: replication and extension. *Psychophysiology* 38 383–390. 10.1111/1469-8986.3830383 11352126

[B2] Babson K. A. Feldner M. T. (2010). Temporal relations between sleep problems and both traumatic event exposure and PTSD: a critical review of the empirical literature. *J. Anxiety Disord.* 24 1–15. 10.1016/j.janxdis.2009.08.002 19716676 PMC2795058

[B3] Bastien C. H. Vallières A. Morin C. M. (2001). Validation of the Insomnia Severity Index as an outcome measure for insomnia research. *Sleep Med.* 2 297–307. 10.1016/s1389-9457(00)00065-4 11438246

[B4] Beck A. T. Ward C. H. Mendelson M. Mock J. Erbaugh J. (1961). An inventory for measuring depression. *Arch. Gen. Psychiatry* 4 561–571. 10.1001/archpsyc.1961.01710120031004 13688369

[B5] Ben-Shakhar G. (1985). Standardization within individuals: a simple method to neutralize individual differences in skin conductance. *Psychophysiology* 22 292–299. 10.1111/j.1469-8986.1985.tb01603.x 4011799

[B6] Boynton G. Vahabzadeh A. Hammoud S. Ruzicka D. L. Chervin R. D. (2013). Validation of the STOP-BANG questionnaire among patients referred for suspected obstructive sleep apnea. *J. Sleep Disord. Treat. Care* 2 1–20. 10.4172/2325-9639.1000121 24800262 PMC4008971

[B7] Braithwaite J. J. Watson D. G. (2015). Issues Surrounding the Normalization and Standardisation of Skin Conductance Responses (SCRs). Technical Research Note. Birmingham: Selective Attention & Awareness Laboratory (SAAL), Behavioural Brain Sciences Centre, School of Psychology, University of Birmingham©.

[B8] Bryant R. A. Creamer M. O’Donnell M. Silove D. McFarlane A. C. (2010). Sleep disturbance immediately prior to trauma predicts subsequent psychiatric disorder. *Sleep* 33 69–74. 10.1093/sleep/33.1.69 20120622 PMC2802249

[B9] Buysse D. J. Reynolds C. F. III Monk T. H. Berman S. R. Kupfer D. J. (1989). The Pittsburgh Sleep Quality Index: a new instrument for psychiatric practice and research. *Psychiatry Res.* 28 193–213. 10.1016/0165-1781(89)90047-4 2748771

[B10] Colvonen P. J. Straus L. D. Acheson D. Gehrman P. (2019). A review of the relationship between emotional learning and memory, sleep, and PTSD. *Curr. Psychiatry Rep.* 21 1–11. 10.1007/s11920-019-0987-2 30661137 PMC6645393

[B11] Concina G. Cambiaghi M. Renna A. Sacchetti B. (2018). Coherent activity between the prelimbic and auditory cortex in the slow-gamma band underlies fear discrimination. *J. Neurosci.* 38 8313–8328. 10.1523/JNEUROSCI.0540-18.2018 30093537 PMC6596172

[B12] Concina G. Renna A. Grosso A. Sacchetti B. (2019). The auditory cortex and the emotional valence of sounds. *Neurosci. Biobehav. Rev.* 98 256–264. 10.1016/j.neubiorev.2019.01.018 30664888

[B13] Concina G. Renna A. Milano L. Manassero E. Stabile F. Sacchetti B. (2021). Expression of IGF-2 receptor in the auditory cortex improves the precision of recent fear memories and maintains detailed remote fear memories over time. *Cereb. Cortex* 31 5381–5395. 10.1093/cercor/bhab165 34145441

[B14] Cornsweet T. N. (1962). The staircase-method in psychophysics. *Am. J. Psychol.* 75 485–491. 10.2307/1419876 13881416

[B15] Cox R. C. Olatunji B. O. (2016). A systematic review of sleep disturbance in anxiety and related disorders. *J. Anxiety Disord.* 37 104–129. 10.1016/j.janxdis.2015.12.001 26745517

[B16] Davidson P. Carlsson I. Jönsson P. Johansson M. (2015). Sleep and the generalization of fear learning. *J. Sleep Res.* 25 88–95. 10.1111/jsr.12339 26359128

[B17] Davidson P. Carlsson I. Jönsson P. Johansson M. (2018). A more generalized fear response after a daytime nap. *Neurobiol. Learn. Mem.* 151 18–27. 10.1016/j.nlm.2018.03.005 29551602

[B18] Deutsch D. (1970). Tones and numbers: specificity of interference in immediate memory. *Science* 168 1604–1605. 10.1126/science.168.3939.1604 5420547

[B19] Diekelmann S. Born J. (2010). The memory function of sleep. *Nat. Rev. Neurosci.* 11 114–126. 10.1038/nrn2762 20046194

[B20] Diekelmann S. Wilhelm I. Born J. (2009). The whats and whens of sleep-dependent memory consolidation. *Sleep Med. Rev.* 13 309–321. 10.1016/j.smrv.2008.08.002 19251443

[B21] DiFazio L. E. Fanselow M. Sharpe M. J. (2022). The effect of stress and reward on encoding future fear memories. *Behav. Brain Res.* 417:113587. 10.1016/j.bbr.2021.113587 34543677 PMC11164563

[B22] Drosopoulos S. Wagner U. Born J. (2005). Sleep enhances explicit recollection in recognition memory. *Learn. Mem.* 12 44–51. 10.1101/lm.83805 15687230 PMC548495

[B23] Dunsmoor J. E. Paz R. (2015). Fear generalization and anxiety: behavioral and neural mechanisms. *Biol. Psychiatry* 78 336–343. 10.1016/j.biopsych.2015.04.010 25981173

[B24] Dunsmoor J. E. Kroes M. C. Braren S. H. Phelps E. A. (2017). Threat intensity widens fear generalization gradients. *Behav. Neurosci.* 131 168–175. 10.1037/bne0000186 28221081 PMC5354976

[B25] Dymond S. Dunsmoor J. E. Vervliet B. Roche B. Hermans D. (2015). Fear generalization in humans: systematic review and implications for anxiety disorder research. *Behav. Ther.* 46 561–582. 10.1016/j.beth.2014.10.001 26459838

[B26] Fernandes-Santos L. Patti C. L. Zanin K. A. Fernandes H. A. Tufik S. Andersen M. L. (2012). Sleep deprivation impairs emotional memory retrieval in mice: influence of sex. *Prog. Neuro Psychopharmacol. Biol. Psychiatry* 38 216–222. 10.1016/j.pnpbp.2012.03.014 22521334

[B27] Fischer S. Drosopoulos S. Tsen J. Born J. (2006). Implicit learning–explicit knowing: a role for sleep in memory system interaction. *J. Cogn. Neurosci.* 18 311–319. 10.1162/jocn.2006.18.3.311 16602193

[B28] Gehrman P. Seelig A. D. Jacobson I. G. Boyko E. J. Hooper T. I. Gackstetter G. D. (2013). Predeployment sleep duration and insomnia symptoms as risk factors for new-onset mental health disorders following military deployment. *Sleep* 36 1009–1018. 10.5665/sleep.2798 23814337 PMC3669076

[B29] Genzel L. Spoormaker V. I. Konrad B. N. Dresler M. (2015). The role of rapid eye movement sleep for amygdala-related memory processing. *Neurobiol. Learn. Mem.* 122 110–121. 10.1016/j.nlm.2015.01.008 25638277

[B30] Ghirlanda S. Enquist M. (2003). A century of generalization. *Anim. Behav.* 66 15–36. 10.1006/anbe.2003.2174

[B31] Goldstein A. N. Walker M. P. (2014). The role of sleep in emotional brain function. *Annu. Rev. Clin. Psychol.* 10 679–708. 10.1146/annurev-clinpsy-032813-153716 24499013 PMC4286245

[B32] Goldstein-Piekarski A. N. Greer S. M. Saletin J. M. Walker M. P. (2015). Sleep deprivation impairs the human central and peripheral nervous system discrimination of social threat. *J. Neurosci.* 35 10135–10145. 10.1523/JNEUROSCI.5254-14.2015 26180190 PMC4502254

[B33] Groeger J. A. Zijlstra F. R. Dijk D. J. (2004). Sleep quantity, sleep difficulties and their perceived consequences in a representative sample of some 2000 British adults. *J. Sleep Res.* 13 359–371. 10.1111/j.1365-2869.2004.00418.x 15560771

[B34] Grosso A. Santoni G. Manassero E. Renna A. Sacchetti B. (2018). A neuronal basis for fear discrimination in the lateral amygdala. *Nat. Commun.* 9:1214. 10.1038/s41467-018-03682-2 29572443 PMC5865209

[B35] Hennevin E. Huetz C. Edeline J. M. (2007). Neural representations during sleep: from sensory processing to memory traces. *Neurobiol. Learn. Mem.* 87 416–440. 10.1016/j.nlm.2006.10.006 17178239

[B36] Holt D. J. Boeke E. A. Wolthusen R. P. Nasr S. Milad M. R. Tootell R. B. (2014). A parametric study of fear generalization to faces and non-face objects: relationship to discrimination thresholds. *Front. Hum. Neurosci.* 8:624. 10.3389/fnhum.2014.00624 25249955 PMC4155784

[B37] Hu P. Stylos-Allan M. Walker M. P. (2006). Sleep facilitates consolidation of emotional declarative memory. *Psychol. Sci.* 17 891–898. 10.1111/j.1467-9280.2006.01799.x 17100790

[B38] Johns M. W. (1991). A new method for measuring daytime sleepiness: the Epworth sleepiness scale. *Sleep* 14 540–545. 10.1093/sleep/14.6.540 1798888

[B39] Jovanovic T. Kazama A. Bachevalier J. Davis M. (2012). Impaired safety signal learning may be a biomarker of PTSD. *Neuropharmacology* 62 695–704. 10.1016/j.neuropharm.2011.02.023 21377482 PMC3146576

[B40] Keller T. A. Cowan N. Saults J. S. (1995). Can auditory memory for tone pitch be rehearsed? *J. Exp. Psychol. Learn. Mem. Cogn.* 21 635–645. 10.1037//0278-7393.21.3.635 7602265

[B41] Kobayashi I. Boarts J. M. Delahanty D. L. (2007). Polysomnographically measured sleep abnormalities in PTSD: a meta-analytic review. *Psychophysiology* 44 660–669. 10.1111/j.1469-8986.2007.537.x 17521374

[B42] Kumar S. Joseph S. Gander P. E. Barascud N. Halpern A. R. Griffiths T. D. (2016). A brain system for auditory working memory. *J. Neurosci.* 36 4492–4505. 10.1523/JNEUROSCI.4341-14.2016 27098693 PMC4837683

[B43] Kuriyama K. Soshi T. Kim Y. (2010). Sleep deprivation facilitates extinction of implicit fear generalization and physiological response to fear. *Biol. Psychiatry.* 68 991–998. 10.1016/j.biopsych.2010.08.015 20889142

[B44] LaBar K. S. Cabeza R. (2006). Cognitive neuroscience of emotional memory. *Nat. Rev. Neurosci.* 7 54–64. 10.1038/nrn1825 16371950

[B45] Lerner I. Lupkin S. M. Sinha N. Tsai A. Gluck M. A. (2017). Baseline levels of rapid eye movement sleep may protect against excessive activity in fear-related neural circuitry. *J Neurosci.* 37 11233–11244. 10.1523/JNEUROSCI.0578-17.2017 29061703 PMC6596812

[B46] Lerner I. Lupkin S. M. Tsai A. Khawaja A. Gluck M. A. (2021). Sleep to remember, sleep to forget: rapid eye movement sleep can have inverse effects on recall and generalization of fear memories. *Neurobiol. Learn. Mem.* 180 107413. 10.1016/j.nlm.2021.107413 33609741

[B47] Lissek S. Bradford D. E. Alvarez R. P. Burton P. Espensen-Sturges T. Reynolds R. C. (2013). Neural substrates of classically conditioned fear-generalization in humans: a parametric fMRI study. *Soc. Cogn. Affect. Neurosci.* 9 1134–1142. 10.1093/scan/nst096 23748500 PMC4127021

[B48] Lo J. C. Groeger J. A. Santhi N. Arbon E. L. Lazar A. S. Hasan S. (2012). Effects of partial and acute total sleep deprivation on performance across cognitive domains, individuals and circadian phase. *PLoS One* 7:e45987. 10.1371/journal.pone.0045987 23029352 PMC3454374

[B49] Luckhaupt S. E. Tak S. Calvert G. M. (2010). The prevalence of short sleep duration by industry and occupation in the National Health Interview Survey. *Sleep* 33 149–159. 10.1093/sleep/33.2.149 20175398 PMC2817902

[B50] Macmillan N. A. Creelman C. D. (2004). *Detection Theory: A User’s Guide.* New York, NY: Psychology press.

[B51] Manassero E. Mana L. Concina G. Renna A. Sacchetti B. (2019). Implicit and explicit systems differently predict possible dangers. *Sci. Rep.* 9 1–12. 10.1038/s41598-019-49751-4 31527740 PMC6746769

[B52] Maquet P. (2001). The role of sleep in learning and memory. *Science* 294 1048–1052. 10.1126/science.1062856 11691982

[B53] Marshall A. J. Acheson D. T. Risbrough V. B. Straus L. D. Drummond S. P. (2014). Fear conditioning, safety learning, and sleep in humans. *J. Neurosci.* 34 11754–11760. 10.1523/JNEUROSCI.0478-14.2014 25164670 PMC6608408

[B54] Mayes A. Montaldi D. Migo E. (2007). Associative memory and the medial temporal lobes. *Trends Cogn. Sci.* 11 126–135. 10.1016/j.tics.2006.12.003 17270487

[B55] McGaugh J. L. (2000). Memory–a century of consolidation. *Science* 287 248–251. 10.1126/science.287.5451.248 10634773

[B56] Menz M. M. Rihm J. S. Büchel C. (2016). REM sleep is causal to successful consolidation of dangerous and safety stimuli and reduces return of fear after extinction. *J. Neurosci.* 36 2148–2160. 10.1523/JNEUROSCI.3083-15.2016 26888926 PMC6602040

[B57] Menz M. M. Rihm J. S. Salari N. Born J. Kalisch R. Pape H. C. (2013). The role of sleep and sleep deprivation in consolidating fear memories. *Neuroimage* 75 87–96. 10.1016/j.neuroimage.2013.03.001 23501052

[B58] Montes-Rodríguez C. J. Rueda-Orozco P. E. Prospéro-García O. (2019). Total sleep deprivation impairs fear memory retrieval by decreasing the basolateral amygdala activity. *Brain Res.* 1719 17–23. 10.1016/j.brainres.2019.05.030 31128099

[B59] Natale V. Esposito M. J. Martoni M. Fabbri M. (2006). Validity of the reduced version of the Morningness-Eveningness Questionnaire. *Sleep Biol. Rhythms* 4 72–74. 10.1111/j.1479-8425.2006.00192.x

[B60] Ojala K. E. Bach D. R. (2020). Measuring learning in human classical threat conditioning: translational, cognitive and methodological considerations. *Neurosci. Biobehav. Rev.* 114 96–112. 10.1016/j.neubiorev.2020.04.019 32343982

[B61] Onat S. Büchel C. (2015). The neuronal basis of fear generalization in humans. *Nat. Neurosci.* 18 1811–1818. 10.1038/nn.4166 26571459

[B62] Pace-Schott E. F. Germain A. Milad M. R. (2015). Effects of sleep on memory for conditioned fear and fear extinction. *Psychol. Bull.* 141 835–857. 10.1037/bul0000014 25894546 PMC4486610

[B63] Patti C. L. Zanin K. A. Sanday L. Kameda S. R. Fernandes-Santos L. Fernandes H. A. (2010). Effects of sleep deprivation on memory in mice: role of state-dependent learning. *Sleep* 33 1669–1679. 10.1093/sleep/33.12.1669 21120129 PMC2982737

[B64] Pechmann T. Mohr G. (1992). Interference in memory for tonal pitch: implications for a working-memory model. *Mem. Cogn.* 20 314–320. 10.3758/BF03199668 1508056

[B65] Pedrabissi L. Santinello M. (1989). *Nuova Versione Italiana Dello STAI Forma Y [New Italian Version of the STAI Form Y].* Firenze: Organizzazioni Speciali.

[B66] Phelps E. A. LeDoux J. E. (2005). Contributions of the amygdala to emotion processing: from animal models to human behavior. *Neuron* 48 175–187. 10.1016/j.neuron.2005.09.025 16242399

[B67] Rieth C. A. Cai D. J. McDevitt E. A. Mednick S. C. (2010). The role of sleep and practice in implicit and explicit motor learning. *Behav. Brain Res.* 214 470–474. 10.1016/j.bbr.2010.05.052 20553972 PMC2921792

[B68] Smith M. T. McCrae C. S. Cheung J. Martin J. L. Harrod C. G. Heald J. L. (2018). Use of actigraphy for the evaluation of sleep disorders and circadian rhythm sleep-wake disorders: an American Academy of Sleep Medicine clinical practice guideline. *J. Clin. Sleep Med.* 14 1231–1237. 10.5664/jcsm.7230 29991437 PMC6040807

[B69] Soehner A. M. Harvey A. G. (2012). Prevalence and functional consequences of severe insomnia symptoms in mood and anxiety disorders: results from a nationally representative sample. *Sleep* 35 1367–1375. 10.5665/sleep.2116 23024435 PMC3443763

[B70] Spielberger C. D. Gorsuch R. L. Lushene R. Vagg P. R. Jacobs G. A. (1983). *Manual for the State-Trait Anxiety Inventory.* Palo Alto, CA: Consulting Psychologists Press.

[B71] Spoormaker V. I. Gvozdanovic G. A. Sämann P. G. Czisch M. (2014). Ventromedial prefrontal cortex activity and rapid eye movement sleep are associated with subsequent fear expression in human subjects. *Exp. Brain Res.* 232 1547–1554. 10.1007/s00221-014-3831-2 24452776

[B72] Squire L. R. (2004). Memory systems of the brain: a brief history and current perspective. *Neurobiol. Learn. Mem.* 82 171–177. 10.1016/j.nlm.2004.06.005 15464402

[B73] Tempesta D. Socci V. De Gennaro L. Ferrara M. (2018). Sleep and emotional processing. *Sleep Med. Rev.* 40 183–195. 10.1016/j.smrv.2017.12.005 29395984

[B74] Walker M. P. van der Helm E. (2009). Overnight therapy? The role of sleep in emotional brain processing. *Psychol. Bull.* 135 731. 10.1037/a0016570 19702380 PMC2890316

[B75] Weber F. D. Wang J. Y. Born J. Inostroza M. (2014). Sleep benefits in parallel implicit and explicit measures of episodic memory. *Learn. Mem.* 21 190–198. 10.1101/lm.033530.113 24634354 PMC3966543

[B76] Wright K. M. Britt T. W. Bliese P. D. Adler A. B. Picchioni D. Moore D. (2011). Insomnia as predictor versus outcome of PTSD and depression among Iraq combat veterans. *J. Clin. Psychol.* 67 1240–1258. 10.1002/jclp.20845 22065464

