# Supplementary material for: Sleep Deprivation During Memory Consolidation, but Not Before Memory Retrieval, Widens Threat Generalization to New Stimuli
Source: Front Neurosci. 2022 May 19;16:902925. doi: 10.3389/fnins.2022.902925 (PMC9160568; doi:10.3389/fnins.2022.902925)
Supplement: Supplementary file 1 [file Image_1.pdf]

Supplementary Material

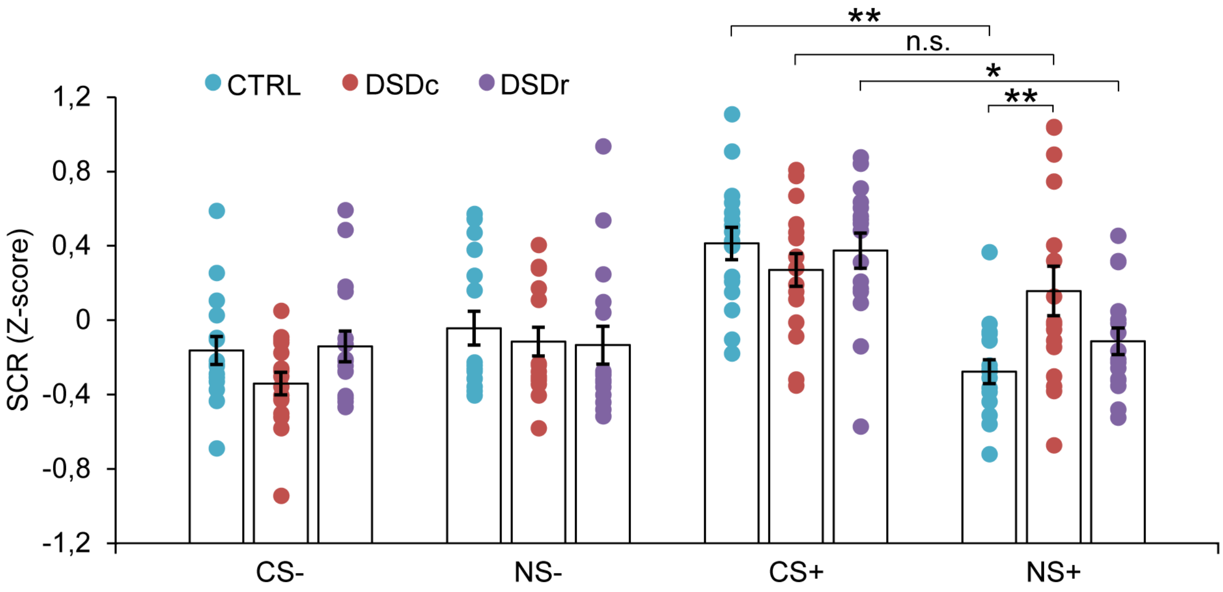

Supplementary Figure 1. Dot plot reporting the effects of DSD on implicit threat memories, safety memories, and threat generalization to new stimuli. Implicit reactions evoked by the learned threatening (CS+) and safety-signaling (CS-) stimuli were comparable among all conditions, whereas implicit reactions triggered by the new stimulus (NS+) were higher in the DSDc group than in the CTRL group. SCRs elicited by the NS+ were weaker relative to those elicited by the CS+ in the CTRL and the DSDr but were similar to those elicited by the CS+ in the DSDc group. * $P < 0.05$, ** $P < 0.01$. All data are mean and SEM. 3×4 mixed ANOVA model followed by Bonferroni corrected simple main effect analyses.
